# A novel iterative mixed model to remap three complex orthopedic traits in dogs

**DOI:** 10.1371/journal.pone.0176932

**Published:** 2017-06-14

**Authors:** Meng Huang, Jessica J. Hayward, Elizabeth Corey, Susan J. Garrison, Gabriela R. Wagner, Ursula Krotscheck, Kei Hayashi, Peter A. Schweitzer, George Lust, Adam R. Boyko, Rory J. Todhunter

**Affiliations:** 1 Department of Crop and Soil Science, Washington State University, Pullman, Washington, United States of America; 2 Department of Biomedical Sciences, College of Veterinary Medicine, Cornell University, Ithaca, New York, United States of America; 3 Cornell Veterinary Biobank, College of Veterinary Medicine, Cornell University, Ithaca, New York, United States of America; 4 Department of Clinical Sciences, College of Veterinary Medicine, Cornell University, Ithaca, New York, United States of America; 5 Sequencing Core, Biotechnology Resource Center, Cornell University, Ithaca, New York, United States of America; 6 Baker Institute for Animal Health, Cornell University, Ithaca, New York, United States of America; 7 Chief Scientific Officer of Embark Veterinary Inc., Austin, Texas, United States of America; Van Andel Institute, UNITED STATES

## Abstract

Hip dysplasia (HD), elbow dysplasia (ED), and rupture of the cranial (anterior) cruciate ligament (RCCL) are the most common complex orthopedic traits of dogs and all result in debilitating osteoarthritis. We reanalyzed previously reported data: the Norberg angle (a quantitative measure of HD) in 921 dogs, ED in 113 cases and 633 controls, and RCCL in 271 cases and 399 controls and their genotypes at ~185,000 single nucleotide polymorphisms. A novel fixed and random model with a circulating probability unification (FarmCPU) function, with marker-based principal components and a kinship matrix to correct for population stratification, was used. A Bonferroni correction at p<0.01 resulted in a P< 6.96 ×10^−8^. Six loci were identified; three for HD and three for RCCL. An associated locus at CFA28:34,369,342 for HD was described previously in the same dogs using a conventional mixed model. No loci were identified for RCCL in the previous report but the two loci for ED in the previous report did not reach genome-wide significance using the FarmCPU model. These results were supported by simulation which demonstrated that the FarmCPU held no power advantage over the linear mixed model for the ED sample but provided additional power for the HD and RCCL samples. Candidate genes for HD and RCCL are discussed. When using FarmCPU software, we recommend a resampling test, that a positive control be used to determine the optimum pseudo quantitative trait nucleotide-based covariate structure of the model, and a negative control be used consisting of permutation testing and the identical resampling test as for the non-permuted phenotypes.

## Introduction

Canine hip dysplasia (HD) is a common complex trait affecting dogs of all sizes, with frequencies ranging from 1–75% [1], and results in painful osteoarthritis (OA). The incidence and severity of HD in the USA over the last 45 years may have declined slightly [2, 3]. The Norberg angle, a quantitative measure of acetabular coverage of the femoral head, is highly correlated with the traditional hip score accorded by the Orthopedic Foundation for Animals (OFA) [1]. Pedigree-based heritability estimates for HD range from 0.2–0.6 [4]. Developmental dysplasia of the human hip has a genetic component and shares phenotypic characteristics with HD [5]. Multipoint linkage analysis and several genome wide association studies (GWAS) for HD collectively suggest that at least 5–10 quantitative trait loci (QTL) control HD expression [6–9]. One gene, *FBN2*, has been directly associated with a hip laxity measurement, the distraction index of HD [10]. Recent canine HD association studies have investigated large samples of mixed and purebred dogs, including Bernese Mountain dogs [7], Dutch and English Labrador Retrievers [8], and German Shepherd dogs [9]. The current study utilized sample genotypes and phenotypes from our previously published study [11].

Elbow dysplasia (ED) in dogs is a group of disorders that affect the articular surfaces of the elbow. The three common abnormalities of ED are fragmented medial coronoid process (FCP), osteochondritis dissecans (OCD) of the medial humeral condyle, and ununited anconeal process (UAP). Adolescent children can develop OCD in the elbow [12]. In Labrador Retrievers, heritability of elbow OA secondary to either OCD or FCP was estimated at 0.27 [13, 14]. When both OCD and FCP were considered together, heritability was as high as 0.77 and 0.45, for Labrador and Golden Retrievers, respectively [15]. More recent estimates put heritability for ED as high as 0.6 for FCP in German Shepherd dogs and at 0.17 in a bivariate analysis for both HD and ED across more than one million purebred dogs in the OFA public registry [3].

Rupture of the cranial (anterior) cruciate ligament (RCCL) is the most common cause of acute and chronic, clinically-severe, hind limb lameness in dogs. The majority of dogs with RCCL lack a history of distinct trauma similar to the non-contact type of human anterior cruciate ligament rupture. Up to 50% of dogs affected with RCCL may have bilateral ruptures [16]. Up to one third of dogs admitted to USA Veterinary Teaching Hospitals for treatment of HD suffer from simultaneous RCCL[16]. Stifle instability following RCCL results in debilitating OA and meniscal injury is a common sequela [17].

Moderate support for genetic susceptibility to non-contact rupture of the anterior cruciate ligament in humans and a predisposition in female athletes has been presented [17]. Rupture of the cranial cruciate ligament in dogs is a complex trait [18] with a heritability of 0.15–0.27 reported in the Newfoundland breed [19, 20]. Four putative QTL were described that underlie RCCL in this Newfoundland pedigree [16]. A GWAS conducted on these same Newfoundland dogs reported associations on CFA1, 10, and 33 [21], none of which overlapped with the loci previously reported by linkage analysis in the same pedigree [22]. However, single nucleotide polymorphisms (SNPs) in key genes involved in ligament strength, stability and extracellular matrix composition were associated with RCCL susceptibility across a single case and a single control from four different dog breeds [21].

Herein, we report the power of a novel fixed and random model circulating probability unification function (FarmCPU), by reanalyzing the genotypes and phenotypes from previous GWAS of HD, ED, and RCCL [11], and compared its power to the standard mixed linear model [23], with simulation, on the same data.

## Materials and methods

### Phenotyping and genotyping

Dogs were admitted to the Cornell University Hospital for Animals for diagnosis of lameness or for genetic screening programs for HD and ED. HD was assessed by the Norberg angle (NA), which measures the relationship between the femoral head and acetabulum on the traditional hip-extended, ventrodorsal radiographic projection. The pelvis of each dog was radiographed following physical maturity. The NA was measured on the Hospital patients by RJT and at the Orthopedic Foundation for Animals by Dr. Gregory Keller. S1 Fig shows radiographic images of an unaffected and a dysplastic dog. The included angle between the geometric centers of each femoral head and the craniodorsal acetabular rim is the NA. The NA ranges up to 120 degrees for a dog with excellent hip conformation and maximum femoral head coverage, to below 50 degrees for a luxated hip and severe HD with secondary osteoarthritis. For GWAS, Norberg angles below 75° were truncated to 75° to approximate a normal distribution.

For ED, the diagnosis was based on elbow radiography, computed tomography and exploratory arthrotomy or arthroscopy performed at the Cornell University Hospital for Animals. Ninety-five percent of the dogs had fragmented medial coronoid process (FCP), some had osteochondrosis (OC) or both FCP and OC, and a few had UAP. The controls were over 2 years of age and diagnosed by elbow radiography either at the Cornell Hospital or by the OFA according to routine phenotypic screening for ED. The OFA uses a flexed lateral radiograph taken at 2 years of age or older, and radiologists look for signs of secondary OA, which is a natural sequela to ED (S1 Fig).

Clinical signs of RCCL included lameness in the affected limb, a positive cranial drawer sign, cranial tibial thrust, synovial effusion, and the presence of a medial proximal tibial buttress. Suspected cases had their stifle(s) radiographed and most of the dogs had arthroscopy or arthrotomy prior to definitive surgical correction. Control dogs were classed as 1 and two classes of affected dogs were used; incomplete ruptures of the ligament (score 2) and complete ligament rupture (score 3). Controls for this trait were over 8 years of age and selected based on orthopedic examination including gait examination, and specifically palpating for normal stifle architecture, stifle stability (negative cranial drawer or cranial tibial thrust) and/or stifle radiography (S1 Fig).

One hundred and twenty of the Labrador Retrievers were over 8 years old and were specifically recruited to be used as controls for mapping inherited traits. To test coat color association as a positive control, among 642 Labrador Retrievers (185 of which were represented in this study) were 264 black, 126 chocolate, and 52 yellow dogs in the Cornell Veterinary Biobank. All Labradors are fixed at the K locus. They are yellow if they have two copies of the recessive allele of *MC1R*, and they are black if they have one or two copies of one of the dominant *MC1R* alleles [24, 25]. The *MC1R* gene is located at CFA5:66,692,398–66,693,344. Color association testing with *MC1R* was performed on a binary trait defined as yellow versus the others (black or chocolate). The tyrosine related protein 1 (*TYRP1)* gene, located at CFA11:33,317,110–33,336,030, causes chocolate coat color in black dogs so that when GWAS was performed on a binary trait defined as black versus chocolate, the marker at CFA11:33,326,685 identified the association [26].

DNA was extracted from venous blood collected in Na-EDTA using either phenol-chloroform or the Puregene (Gentra) protocol. All the dogs were genotyped for 185,805 SNPs on the Illumina semi-customized version 2 of the canine HD mapping array [11]. Genotype and phenotype data were deposited in Dryad (doi:10.5061/dryad.266k4).

#### Statistical analysis

Before statistical analysis, SNPs with minimum allele frequency (MAF) < 0.05 were removed. The top five principal components were included in the model to account for breed effect and population structure in all three traits. The statistical modeling was done with FarmCPU [27], which combines the advantages of both the fixed and random effects model implemented in R, and which tests the significance of the additive genetic effect by the standard *t* test. The bin method was employed to avoid the confounding problem of linkage disequilibrium (LD). The default setting of bin size was used in HD, ED and RCCL association testing.

**Fig 1:**
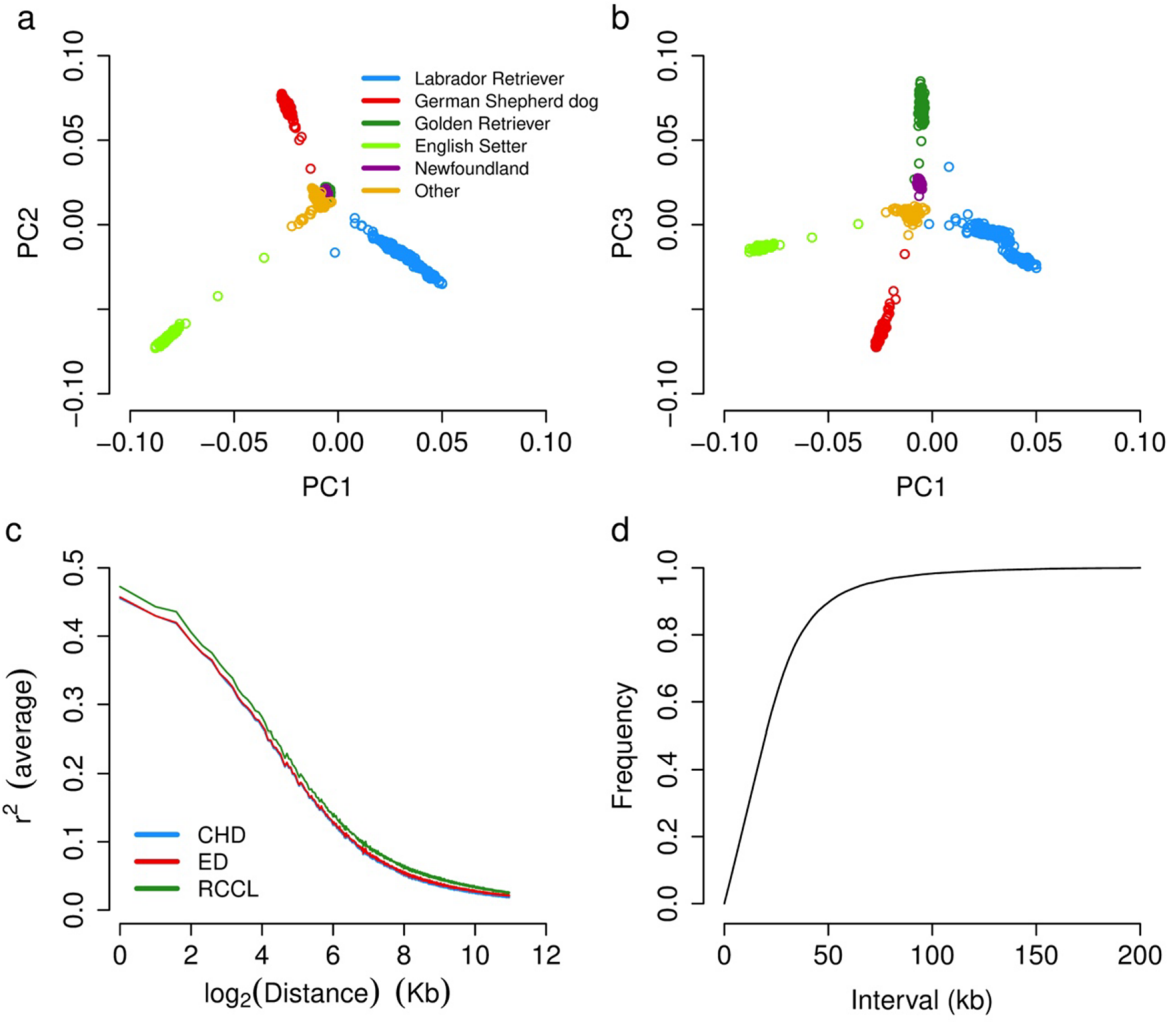
The summary of genetic relationships among the breeds with the most individuals and the decay of linkage disequilibrium (LD). Along the top three principal components of variation, the main breeds are genetically distinct (a, b). The extent of LD, measured as average pair-wise r^2^, drops below 0.3 at 11 kb in multiple populations (c). The marker density plot shows the distribution of marker intervals and 80% of marker intervals are less than 30 kb (d). The genotype data used for principal component calculation and genetic relationship building comes from the biggest dataset for HD [11].

**Fig 2:**
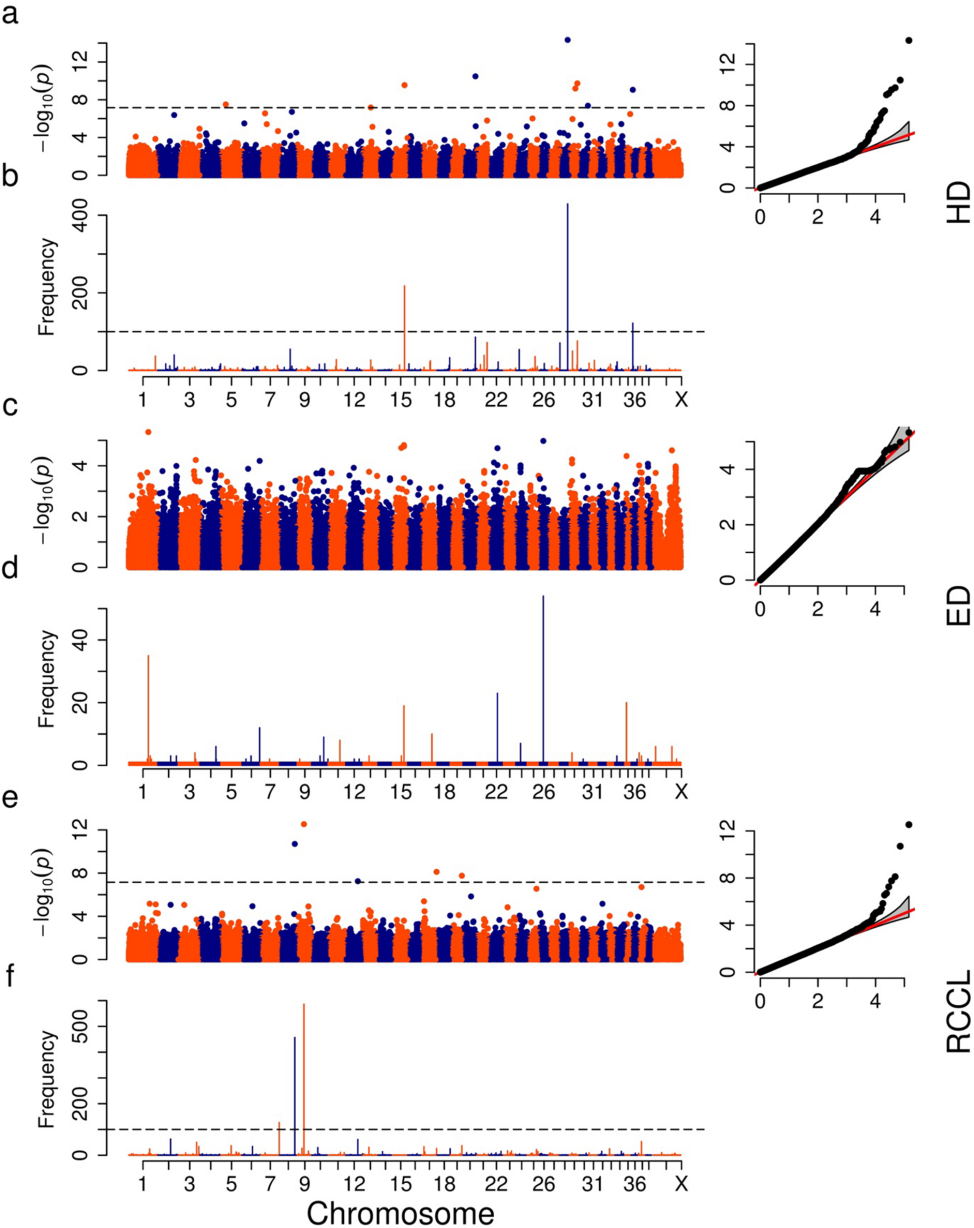
**Manhattan plots, QQ plots and resampling test plots of GWAS for hip dysplasia (HD), elbow dysplasia (ED) and rupture of the cranial cruciate ligament (RCCL) (a, c and e)**. The dashed horizontal line depicts the Bonferroni-adjusted significance threshold (genome wide α level of 0.01). The QQ plot of association with the null distribution (expected) versus the observed associations is shown as insert. The Manhattan plot of the resampling test (b, d, and f) shows the y axis indicating the number of significant occurrences or associations [expressed as the–log(p)] in 1,000 replicated tests across the genome. Note the scale along the Y axis for the associated–log(p) value varies by plot.

The principle component analysis and LD testing in Fig 1 were calculated using PLINK [28] (Fig 2 and S2 Fig) were generated by the Genome Association and Prediction Integrated Tool (GAPIT) [29]. A resampling test was used to identify false positive signals through selection of 80% of the individuals without replacement to conduct the GWAS and the process was repeated 1,000 times for all the dogs. The occurrence of any SNP whose *P*-value passed the Bonferroni cutoff (P < 0.01) was counted and 100 of 1000 repeats was used as the criterion of a false positive. For false negative testing of the resampling test, the phenotypes were shuffled relative to the genotypes and 100 pseudo phenotype datasets were generated. Then, the resampling test was implemented exactly as for the real data relationships. For false negative testing of the whole population GWAS, the phenotypes were shuffled and 1000 pseudo phenotype datasets were generated. For the false negative threshold, the same criterion was applied i.e. when the cut off was 100 of the 1,000 replicates, the FDR for the permutation test was <0.05, so we used 100 as the cut off.

In the simulation test, genotype data of all samples was employed to simulate the phenotype value in the three populations (HD, ED, or RCCL). The phenotype value included the additive genetic effect and the residual effect, and both of them follow the normal distribution. The heritability of the three simulated phenotypes was 0.75 (HD), 0.25 (ED) and 0.75 (RCCL), respectively. Heritability based on marker information that was calculated in all three traits was generated from GAPIT [29]. For all three simulated traits, 50 causal SNPs were randomly selected from all the markers. Fifty causal SNPs were simulated as a reasonable approximation to the unknown real number because the genetic effect is due to the additive effect of many loci each of small effect. The simulation was repeated 100 times to calculate statistical power and false discovery rate (FDR). When we calculated the power of each model and its FDR, all the SNPs within each replicate of the simulation were ranked by P value. The power was calculated as: Power = N_Q_ / N_QT_, and FDR was calculated as: FDR = N_F_ / (N_QT_ + N_F_), where N_Q_ is number of true positive associations, N_QT_ is total number of QTN, and N_F_ is total number of false positives. The power and FDR calculation were conducted across all the QTNs. All the SNPs within a fixed range around each QTN were treated as loci in the same LD interval (true positives), and the window size of this range was set as 100,000 bp. Simulated phenotypes were generated using GAPIT [29]. The mixed linear model was performed in GEMMA [23] and compared to analysis of the same simulated phenotype data using FarmCPU.

## Results

### Marker and breed statistics

The Norberg angle (101.8±SD = 9.8°) was averaged over both hips of 921 dogs belonging to 69 pure and 121 mixed breeds. Trait values of the five breeds with the most individuals represented are shown in S1 Table. The presence or absence of ED was assessed, as described in the Methods Section, on 746 dogs of 82 pure and 20 mixed breeds (113 cases and 633 controls). Clinical phenotyping of RCCL is described in the Methods Section, and GWAS was conducted on 603 dogs across 68 pure and 53 mixed breeds (141 cases with a complete cranial cruciate ligament tear, 84 with a partial tear, and 378 controls) (S1 Table). About half of all the dogs had more than a single phenotype measured; 412 for both HD and RCCL, 440 for both RCCL and ED, 476 dogs for both HD and ED, and 306 dogs for all three phenotypes, and thus could be included as cases or controls for multiple traits.

In total, 180,117 SNPs remained after filtering, with an overall call rate of 99.8% [11]. The relationship between the first three principal components (PCs) of these genotypes indicated that the five breeds with the most individuals represented were genetically discrete (Fig 1A and 1B). The LD decay was rapid reaching an *r*^*2*^ of 0.3 at an average of 11 kb in all three mapping populations (Fig 1C). Marker density across the whole genome was ~1 SNP per 15 kb (Fig 1D).

### Canine hip dysplasia

#### GWAS results and internal validation through resampling

Nine SNPs were significantly associated with HD (Fig 2A) but only three passed the resampling test (Fig 2B). The linear model, that included the top five PCs and possible quantitative trait nucleotides (QTNs) as covariates of the fixed effects, controlled the potential inflationary effects of population stratification according to the quantile-quantile (QQ) plot (Fig 2A). The significantly associated SNP at CFA36:7,366,577 was segregating in the five breeds with the most dogs and the loci on CFA15 and CFA28 were segregating in four of the breeds with the most dogs (S2 Table).

To test the level of confidence in the associated SNPs, the resampling test was applied based on the number of times out of 1,000 times that the association exceeded the Bonferroni cutoff (p<0.01) (Fig 2B). The three significant SNPs on CFA15, 28 and 36 exceeded the resampling test threshold more than 100 times. This was the significance threshold based on the negative control results and applying identical criteria as to the original relationships (Fig 2B).

#### Candidate genes for HD

The SNP at CFA28:34,369,342 for the Norberg angle (Table 1) was described previously in the same dogs and is within C-terminal binding protein 2 (*CTBP2*), a transcriptional co-repressor [11]. The marker at CFA15:51,083,415 is closest to tripartite motif-containing protein 2 (*TRIM2*), an E2 ubiquitin ligase (Table 1). The marker at CFA36:7,366,577 is nearest dipeptidyl peptidase 4 (*DPP4*), a membrane bound peptidase previously associated with rheumatoid arthritis and OA (Table 1).

**Table 1:** SNPs associated with hip dysplasia (HD) and rupture of the cranial cruciate ligament (RCCL) and positional candidate genes based on the FarmCPU method.

Trait:Marker	CFA^a^:Position^b^	*P*-value	MAF^c^	Candidate gene	Distance	Frequency^d^
HD: BICF2G630265083	28:34,369,342	4.61E-15	0.07	*CTBP2*	0	429
HD: BICF2S23013075	15:51,083,415	2.86E-10	0.26	*TRIM2*	40 kb	218
HD: BICF2P1242852	36:7,366,577	8.88E-10	0.13	*DPP4*	55 kb	122
RCCL: BICF2G630833273	9:28,692,896	2.93E-13	0.32	-	-	586
RCCL: BICF2S23229897	8:64,204,398	1.98E-11	0.21	*CLMN*	84 kb	457
RCCL: TIGRP2P105607_rs8747452	7:79,570,691	1.02E-03	0.12	*DYN*	100 kb	127

^a^ The chromosome (CFA)

^b^ physical position in base pairs (bp)

^c^ minor allele frequency (MAF)

^d^ frequency of resampling test cross-validation (100 repeats is the significance criterion based on positive and negative resampling tests).

### Elbow dysplasia

#### GWAS results and resampling test validation

Among the 746 dogs genotyped for ED, 79 breeds were represented (S1 Table). No marker association passed the Bonferroni-adjusted genome wide p value (Fig 2C) even though the identical two loci that were associated with ED in the previous published analysis [11] were associated with ED most often in the resampling test (Fig 2D).

### Ruptured cranial cruciate ligament

#### GWAS results and resampling test validation

Five SNPs exceeded the genome wide threshold in the primary GWAS that included all the dogs (Fig 2E). However, only three of these passed the resampling test (Fig 2F). Marker position and candidate genes are shown in Table 1. The SNP at CFA7:79,570,691 did not pass the genome wide threshold for the primary GWAS but did exceed the 100 times threshold in the resampling test (Fig 2F). The three significantly associated SNPs in the GWAS were segregating in Labrador Retriever, Golden Retriever and German Shepherd dogs (S2 Table).

We reanalyzed the RCCL trait using the GEMMA [23] mixed model as described in Hayward et al. [11]. Separation of the partial and complete tears within the case group revealed the same association on CFA9:28,692,896 with p = 1.87x10^-7^. This association would have been significant at the Hayward et al [11] Bonferroni-adjusted threshold at α < 0.05 of p = 3.5x10^-7^. With the cases categorized into 2 classes (partial or complete), the association on CFA8:64,204,398 was below the Bonferroni-adjusted threshold in the GEMMA-based analysis with p = 7.5x10^-6^. There was no association on CFA7 in the GEMMA-based analysis.

#### Candidate genes for RCCL

No candidate genes were in proximity to the associated marker on CFA9. The closest candidate gene to the marker at CFA8:64,204,398 is calmin (C*LMN*), a brain protein expressed during development (Table 1). The candidate gene closest to the marker at CFA7:79,570,691 is dymeclin (*DYN*) (Table 1), which encodes a Golgi-related protein involved in secretory pathways essential to endochondral bone formation during early development.

#### Positive control and negative control

Coat color in Labrador Retriever dogs was employed as the positive control to determine the optimal covariate structure needed for the FarmCPU method. When GWAS was performed on a binary trait defined as yellow versus the others (black or chocolate), the SNP at CFA5:63,694,334 had the strongest association signal and is located within the melanocortin 1 receptor (*MC1R*) as expected [24, 25] (S2 Fig). The tyrosine related protein 1 (*TYRP1)* gene, located at CFA11:33,317,110–33,336,030 causes chocolate coat color in black dogs so that when GWAS was performed on a binary trait defined as black versus chocolate, the marker at CFA11:33,326,685 was strongly associated as expected (S2 Fig) [26].

The negative control tests were conducted in two ways. One was performed to determine the significant cutoff for the resampling test; the other was the permutation test for the whole population in the GWAS. For the resampling test, if 100 of 1000 repeats was employed as the cutoff, the resampling test would pass the permutation test significant level of 0.05 on all three traits. For the whole population permutation test, the false positive proportion were 1.2% (HD), 0.8% (ED) and 0% (RCCL) respectively, which are all lower than the significance criterion of 5%.

#### Simulation test

The statistical power difference of FarmCPU and the mixed linear model was evaluated through a simulation test in the three populations. The heritability of these three traits was 97.1% (HD), 20.4% (ED) and 100% (RCCL) respectively, which was calculated using the genotypes. Thus the heritability of HD and RCCL in current population sample is much higher than in the natural population. Accordingly, the heritability of the simulated phenotype was set as 75% (HD), 25% (ED) and 75% (RCCL), respectively. The power of FarmCPU was better than the mixed linear model in the HD and RCCL population samples and slightly worse than mixed linear model in the ED sample. These results coincided with the respective model analyses of the real traits. Under the scenario of low heritability, FarmCPU will be identical or similar to GLM because few of the possible QTNs could be captured and treated as fixed effects in the model. As such, the statistical power of MLM may be higher than FarmCPU in those traits with low heritability in a GWAS population sample. The breeds, phenotypes, and genotypes are in accompanying S3 Table.

## Discussion

### Association analysis of SNP-phenotype

A GWAS of these three orthopedic traits on this population of dogs using the same genotypes and phenotypes based on a linear mixed mapping model implemented in GEMMA v. 0.94 has been previously reported [11, 23]. Here we analyzed the same data with a novel statistical software, FarmCPU [27]. Based on this model, we confirmed the previous locus associated with HD [11], failed to confirm the previous associations with ED in the same dogs at the preset genome-wide p value threshold, and report an additional two loci that were associated with HD and three associated with RCCL. However, when we reanalyzed the GWAS encoding RCCL as a semi-quantitative trait with three levels (two types of cases versus controls) using a linear mixed model in GEMMA [23], the same model used in Hayward *et al*.[4], we did uncover the marker association on CFA9 with RCCL at the Bonferroni-adjusted genome wide level of significance used in Hayward *et al*. [11]. Our confidence in this total number of associations is supported by the number of successful replicates in the resampling test (cross validation) that surpassed the Bonferroni-adjusted threshold of 100 successful replicates based on the permutation testing.

False positives in a GWAS can be effectively controlled by a fixed and a random effect mixed linear model that incorporates population structure and kinship among individuals to adjust the effect of a marker association. However, the adjustment for occult population structure can penalize the number of true positive associations. The FarmCPU approach extends previous mixed linear models into two separate stages. The fixed effect component tests each SNP, one at a time, with multiple associated markers (pseudo quantitative trait nucleotides) as covariates to control false positive associations. To avoid model over-fitting, the effect of the associated markers is then estimated as a random effect in the second stage by using them to define kinship. Previously, both real and simulated data analyses demonstrated that FarmCPU improved statistical power compared to current methods, while still controlling inflation [27]. Here we tested the power of the two methods by simulation using the three samples for HD, ED, and RCCL. That result supported the previous report by Liu et al. [27] using plant and human data, which we now extend to canine samples showing power advantages of the FarmCPU method for mapping of HD and RCCL. The ED sample was heavily weighted toward control dogs and shows that each sample and population structure will influence the power of each model.

It is important when mapping complex traits in veterinary medicine to maximize power, as proffered through the FarmCPU software, because financial constraints limit the number of individuals that can be phenotyped and genotyped for GWAS. Yet, we remained concerned about controlling false positive associations and so we set the Bonferroni-adjusted, genome wide p value to <0.01 instead of the standard <0.05 level. When only three PCs were used as covariates in the model, we found 2 spurious associations with coat color yet the QQ plot showed a lack of inflation in p values in the lower part of the curve (data not shown). By using five PCs as covariates, the spurious associations were removed (S2 Fig). A second important note relates to the number of replicates that pass the genome wide threshold in the resampling test. We used 1,000 replicates in the resampling test and set the threshold of a successful association at a Bonferroni adjusted genome wide p value of < 0.01, just as in the original GWAS using all the dogs. One hundred out of 1,000 replicates were used as the minimum number to support the association based on the permutation testing (Table 1).

The validity of these associations requires replication in an independent sample of pure and mixed breed dogs and sequencing of the candidate genes [9]. Hayward *et al*. [11], demonstrated by simulation that 500–1,000 cases and 500–1,000 controls across breeds should be genotyped and analyzed concurrently to detect causal loci of moderate effect based on a GEMMA-based (mixed linear) model [11]. Here, we gathered the minimum predicted number of dogs belonging to many breeds but lacked power within more than about five breeds to detect segregation of the markers. This is a drawback of a mapping population where the phenotype is based on a diagnosis made in a hospital setting. The diagnostic phenotyping is likely to be correct but the sample of the population is governed by the randomness of the patients admitted to a hospital. A further drawback of hospital-based sampling is the difficulty of acquiring a control group because healthy dogs are not admitted to a hospital. In our case, some of the controls were deliberately recruited, were available from special closed colonies, or were identified as having some normal joints as part of routine orthopedic screening in the hospital. The advantage of mapping in such populations is that LD is narrower across breeds and thus the search interval for candidate genes is narrower than compared to within-breed mapping (Table 1).

### Candidate genes for HD

Comparative mapping and candidate gene screening of cognate complex traits in dogs and people may lead to discovery of common molecular genetic etiologies. Both the human [30,31] and canine disorders have a heritable component [4]. Common features of the phenotype in both dogs and people include delayed ossification of the femoral head, hip incongruity, a familial tendency, and a later expression of hip OA causing pain and disability [30]. Although several polymorphisms have been associated with developmental dysplasia of the human hip [30–33], only a single gene has been reported that segregates across ethnicities and populations [33]. Comparative mapping between the human and canine disorders may eventually provide mutually supportive evidence for causal genes of each disorder. To date, none of the candidate genes or mutations associated with the human disorder; a chemokine receptor, *CX3CR1* [32], a patterning gene, *HOXB9* [30, 34, 35], or ubiquitin-fold modifier 1-specific peptidase 2 (*UFSP2*) [36] have been associated with the canine orthologue. Nor have any of these associations been independently replicated in human studies.

C-terminal binding protein 2 (*CTBP2*) contains the marker associated with HD at CFA28:34,369,342 in this report and in Hayward et al., [11]. C-terminal binding proteins exert transcriptional repression primarily via recruitment to DNA of a corepressor complex that consists of histone deacetylases and methyltransferases. *Ctbp1*__+_*/-*_*;Ctbp2*_-*/-*_ mouse embryos fail to form normal musculoskeletal structures like vertebral bodies, distal ribs, and skeletal muscle [37]. Both *Ctbp1* and *Ctbp2* are expressed in the ventral sclerotomal somitic domain, and later in the myotome and dermatomyotome, that give rise to skeletal muscles and distal ribs [38]. C-terminal binding proteins are corepressors for mediators of wingless integration site (*Wnt*), *Notch*, and bone morphogenetic protein (*Bmp*) signaling. *Wnt*, together with *Bmp* signaling, controls the patterning of the dorsal and ventral muscles in the developing limb bud [39, 40]. Based on our RNA seq, *CtBP1* and *2* are both expressed in canine hip joint capsule and round ligament of the femoral head (data not shown).

The marker associated with HD at CFA15:51,083,415 is ~40 kb from tripartite motif-containing protein 2 (*TRIM2*), an E2 ubiquitin ligase. Mutations in E2 cause Charcot-Marie-Tooth disease type 2R characterized by muscle weakness, atrophy, and axonal degeneration [41]. As early as 8 weeks of age, Cardinet *et al*. [42] described decreased type II fiber type in the pectineus muscle, and reduced pelvic muscle mass, in dogs which subsequently developed HD, when compared to control dogs. Pelvic muscle strength and coordination are critical players for hip stabilization. Finally, breeds with high muscle to bone mass ratio, like the Greyhound and Whippet, are less susceptible to HD [1].

The marker at BICF2P1242852 on CFA7 is ~55 kb from dipeptidyl peptidase-4 (*DPP4*), which is a plasma membrane peptidase that selectively cleaves an N-terminal dipeptide from peptides with a proline or alanine residue [43]. Dipeptidyl peptidase-4 is widely expressed, and is involved in T cell function [44, 45]. Changes in the expression and/or the blood plasma concentration of *DPP4* are associated with several diseases including rheumatoid arthritis and OA [46]. Dipeptidyl peptidase-4 is highly expressed by adipocytes and resident macrophages/dendritic cells in inflamed adipose tissue from obese subjects [47]. It is unclear how mutations in *DPP4* could influence hip conformation but it is possible that secondary hip OA could be affected by *DPP4* function because its cytokine and growth factor cleavage products will influence tissue inflammation. It is conceivable that separate mutations can influence the primary heritable HD defect and the secondary organ response to the disorder [48, 49].

Previously published studies identified regions on CFA01, CFA8, CFA20, and CFA25 associated with HD in Labrador Retrievers [8]. However, despite our sample population containing 242 Labrador Retrievers, we did not identify any corresponding regions of significant association. Some of their associated loci [8] had linkage support from HD mapping in the breeds used in our report [9, 50–52].

### Candidate genes for RCCL

The closest candidate gene for RCCL in the region of CFA8:64,204,398 is calmin (*CLMN*). Calmin is a developmentally-regulated brain protein with calponin-homology domains [53]. Calmin has non-motor, actin binding function belonging to the spectrin/filamin related cytoskeletal proteins. Genome wide association studies for metabolic disorders including response to statins [54] and psychiatric disorders [55] have identified significant associations with markers in or near CLMN. The evolution of dog domestication has been reflected in underlying genetic changes in metabolic and cognitive function [55] so that the association between CLMN polymorphisms and RCCL may be related to domestication sweeps in the evolution of the domestic dog [56]. Dyggve–Melchior–Clausen syndrome and Smith-McCort dysplasia are recessive spondyloepimetaphyseal dysplasias caused by loss-of-function mutations in dymeclin (*DYN)* [57–59], the candidate gene closest to the marker at CFA7:79,570,691 associated with RCCL. Dymeclin is involved in Golgi trafficking and *DYN* mutations cause delayed endoplasmic reticulum to Golgi trafficking [60]. There has been a long debate as to whether excessive caudal slope of the proximal tibia, that might result from a local dysplasia, places excessive overload on a cranial cruciate ligament susceptible to rupture [61].

Polymorphisms in fibrillin 2 (*FBN2*) [62], vascular endothelial growth factor A (*VEGFA*), kinase insert-domain receptor (*KDR*) [63], *COL1A1* [64], decorin (*DCN*), aggrecan (*ACN*), biglycan (*BGN*), *LUM* (lumican) [65], *COL5A1* [66], and interactions between polymorphisms in *COL5A1* and *COL12A1* have been associated with rupture of the anterior cruciate ligament in people, especially in female athletes, but none of these genes were associated with RCCL in our study.

## Conclusion

Our report is the first to apply FarmCPU, a novel statistical association method, to canine complex trait mapping. We confirmed the previous association on CFA28 with HD and identified 5 further associations. We confirmed a further association using the linear mixed model applied in the Hayward *et al*.[11] analysis for RCCL when we reanalyzed the RCCL phenotype as a semi-quantitative trait. Across-breed mapping enabled us to reduce the shared LD interval and hence the number of candidate genes to screen, in these large animal models of human complex diseases [11, 67]. Simulations demonstrated that FarmCPU had more power than a mixed linear model implemented in GEMMA for mapping complex traits of moderate to high heritability. Although complex orthopedic traits may be associated with several extracellular matrix genes, our current analysis suggests that defects in genetic regulation may also be at play.

## Ethics statement

Cornell Institutional Animal Care and Use Committee approval numbers were 2005–0151 (Cornell Medical Genetic Archive) and 2006–0187 (Hip Dysplasia and Osteoarthritis Genetics).

## Supporting information

S1 Fig (A) Radiographic images of a dog with normal hip joints and a dog which is severely affected with osteoarthritis secondary to hip dysplasia (B). (C) Radiographic images of a dog which has a normal stifle joint and a dog which is severely affected with osteoarthritis secondary to rupture of the cranial cruciate ligament (D). (E) Radiographic images of a dog which has a normal elbow joint and a dog which is severely affected with osteoarthritis secondary to elbow dysplasia (F).
(DOCX)

S2 Fig Manhattan plot of GWAS for coat color in 642 Labrador Retriever dogs (264 black, 126 chocolate, and 52 yellow dogs). Dashed line is Bonferroni adjusted genome wide p < 0.01. (a) When GWAS was performed on a binary trait defined as yellow versus the others (black or chocolate), the SNP at CFA5:63,694,334 had the strongest association signal and is located within *MC1R*. (b) The tyrosine related protein 1 (*TYRP1)* gene, located at CFA11:33,317,110–33,336,030, causes chocolate coat color in black dogs so that when GWAS was performed on a binary trait defined as black versus chocolate, the marker at CFA11:33,326,685 was strongly associated. Note the Y axis scale denoting the strength of the association is different for the two color traits.
(DOCX)

S3 Fig Negative control for mapping hip dysplasia (HD), elbow dysplasia (ED) and rupture of the cranial cruciate ligament (RCCL). For the permutation, the real phenotype data of these three traits was shuffled and, for the resampling test, 80% of the individuals were sampled each time. This process was repeated 1,000 times and the most frequently associated loci were collected and their frequency displayed in the Box Plots (a) and the cumulative distributions for each trait (b, c, d). Black dashed line (b, c, d) refers to the cutoff of 100 that we used in real trait-genotype resample testing. Note the Y axis scale denoting the frequency of each replicated association is different for each panel.
(DOCX)

S4 Fig Power comparison between FarmCPU and MLM in GEMMA based on simulated data described in the Methods Section for in HD, ED and RCCL. The vertical axis is the statistical power of 50 different randomly sampled causal loci and the horizontal axis is the false discovery rate (FDR) of different causal loci. The window size of each locus in both the power and FDR calculations was defined as 100,000 bp.
(DOCX)

S1 Table Phenotype summary across breeds with the most individuals (or the remainder of the dogs) for hip dysplasia, elbow dysplasia, and rupture of the cranial cruciate ligament. Hip dysplasia was measured as the Norberg angle. Elbow dysplasia was scored as present or absent based on physical examination and radiography, computed tomography and/or surgery. Rupture of the cranial cruciate ligament was assessed by physical examination, radiography, and surgery, and scored as control (unaffected, score of 1) or case, which could be a partial rupture (score of 2) or a complete rupture (score of 3) based on surgery.
(DOCX)

S2 Table Segregation of associated SNPs in the breeds with the most dogs for hip dysplasia and rupture of the cranial cruciate ligament. (XLSX)

S3 Table Dog identification, breeds, and phenotypes that were used in the analyses shown in this paper. This data can be linked to the genotypes in Dryad (datadryad.org, doi:10.5061/dryad.266k4) through the common IDs.
(XLSX)
