# Supplementary figures and images for: A novel iterative mixed model to remap three complex orthopedic traits in dogs
Source: PLoS One. 2017 Jun 14;12(6):e0176932. doi: 10.1371/journal.pone.0176932 (PMC5470659; doi:10.1371/journal.pone.0176932)

F

E

D

C

B

A


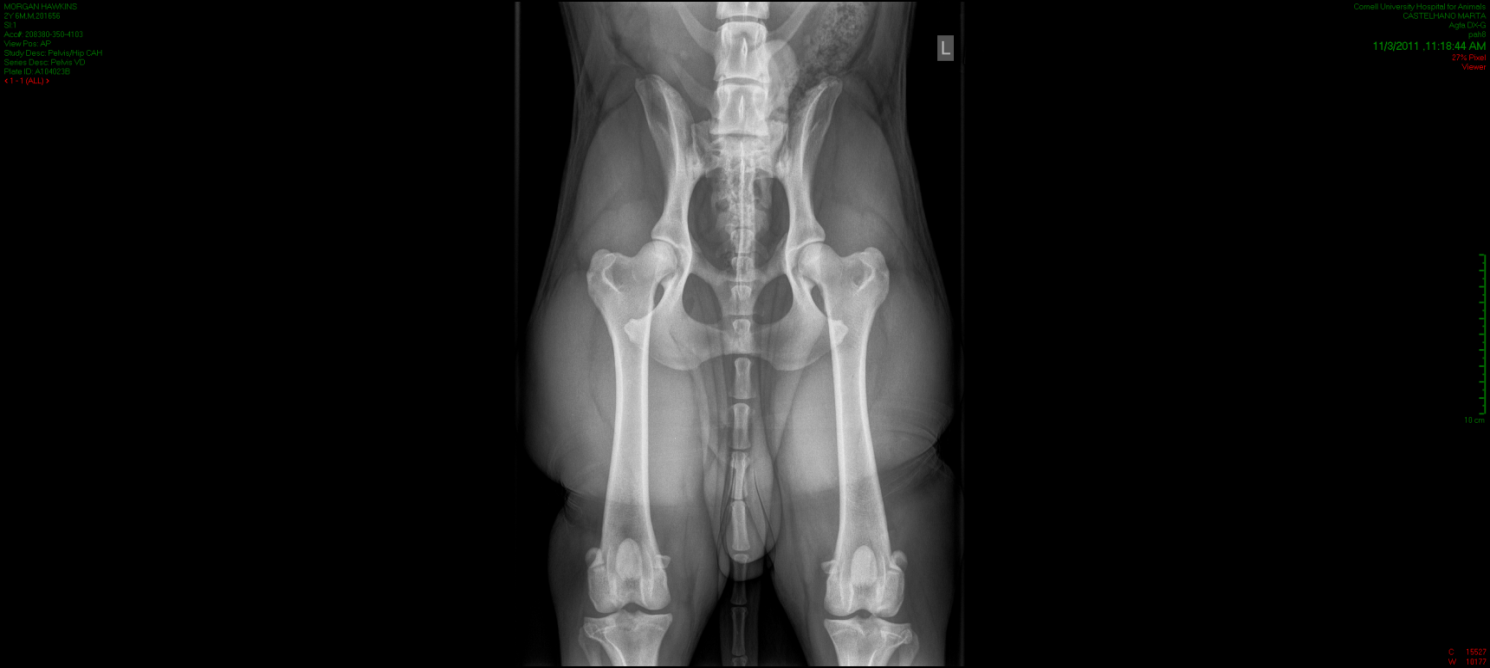

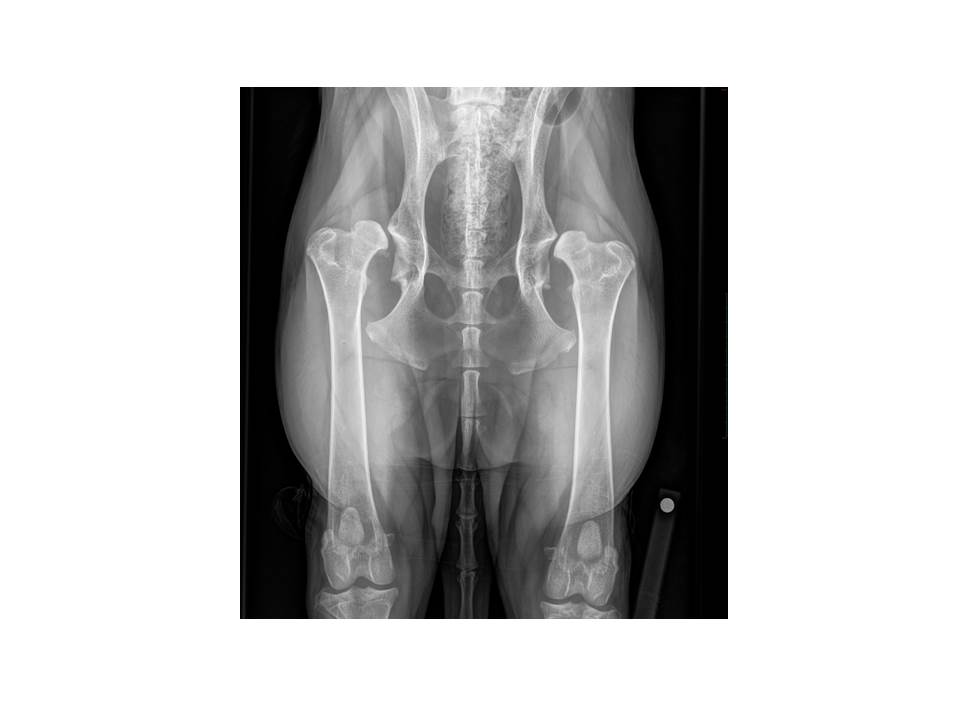

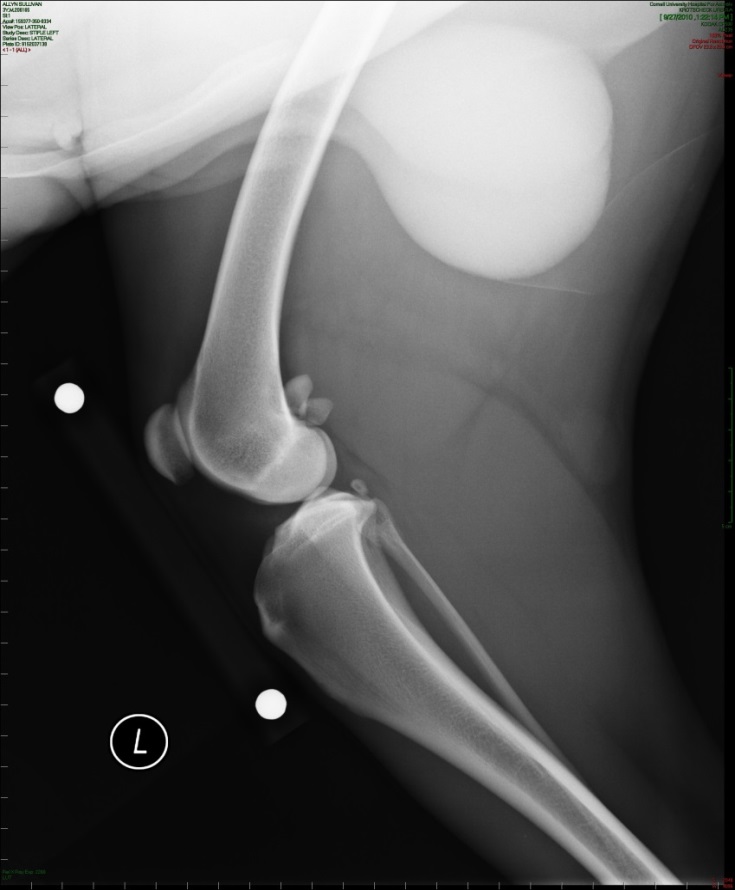

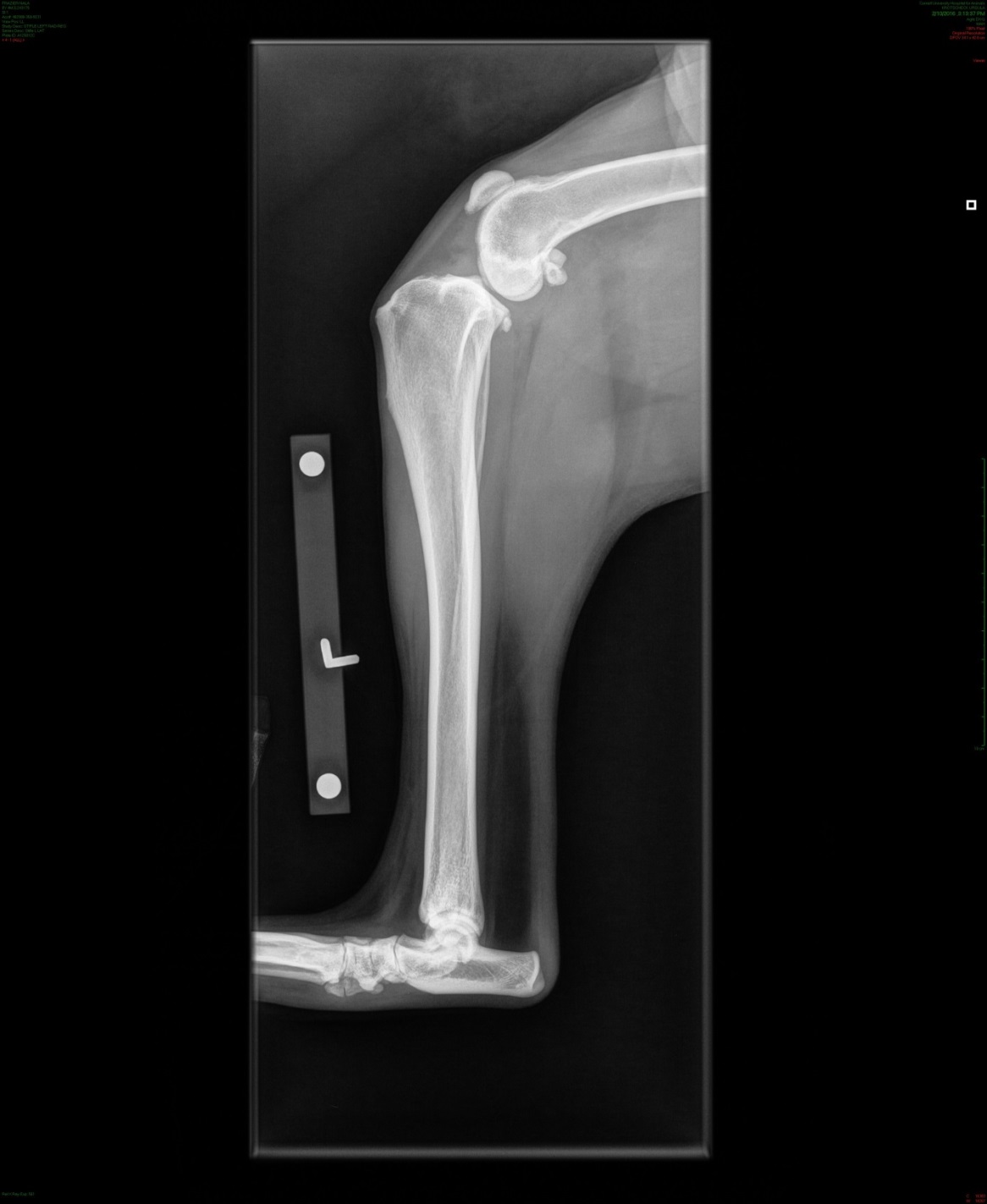

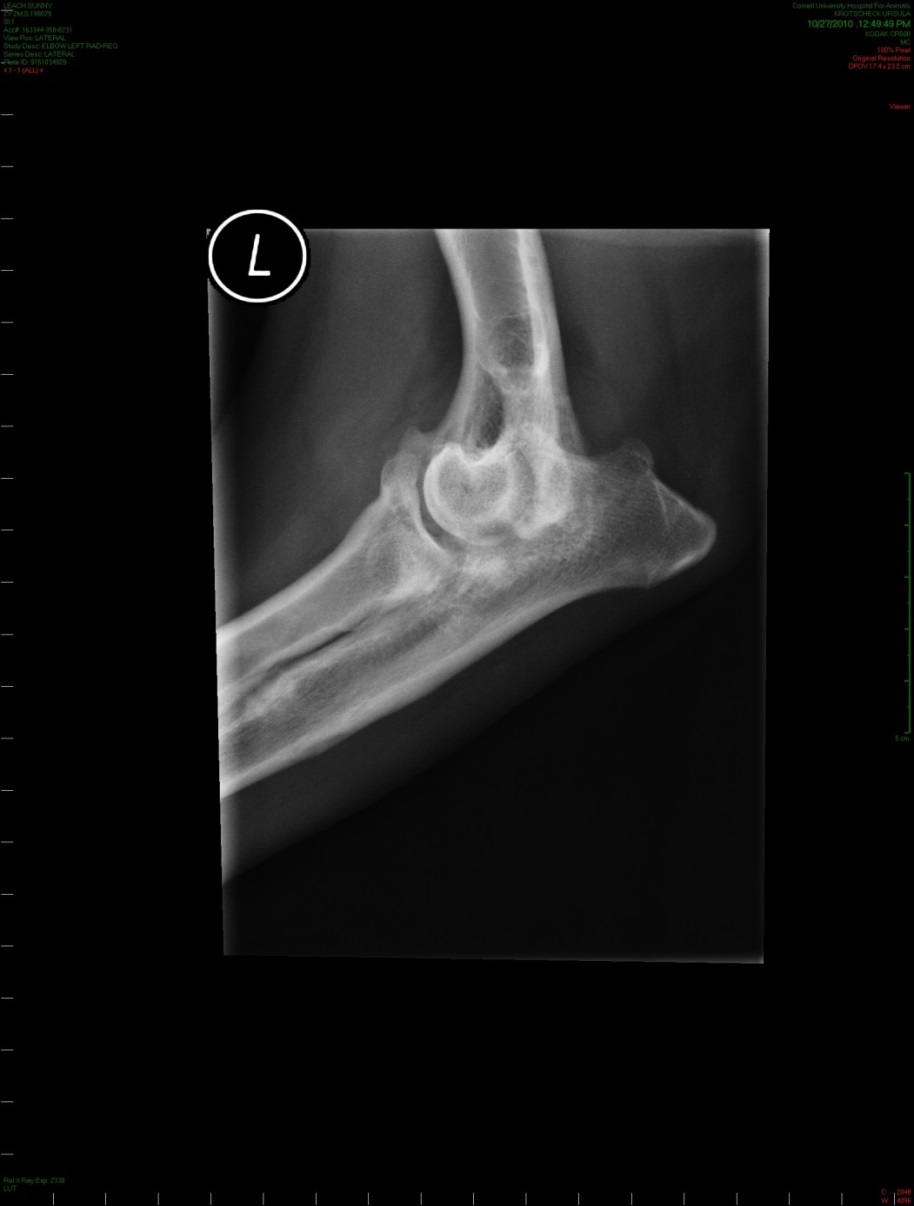

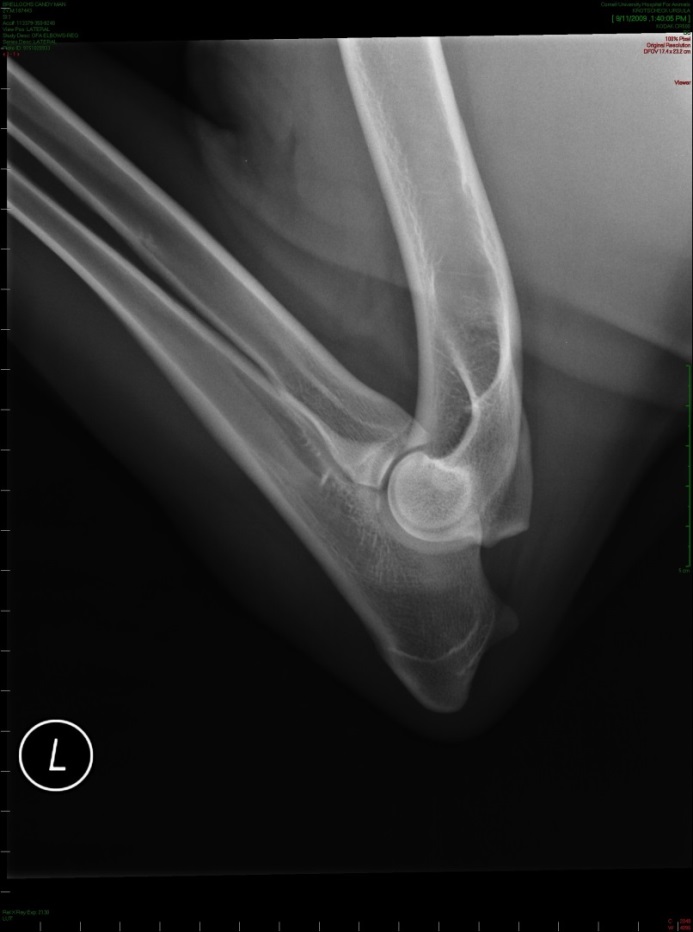

Supplement: S1 Fig — (A) Radiographic images of a dog with normal hip joints and a dog which is severely affected with osteoarthritis secondary to hip dysplasia (B). (C) Radiographic images of a dog which has a normal stifle joint and a dog which is severely affected with osteoarthritis secondary to rupture of the cranial cruciate ligament (D). (E) Radiographic images of a dog which has a normal elbow joint and a dog which is severely affected with osteoarthritis secondary to elbow dysplasia (F). (DOCX) [file pone.0176932.s001.docx]

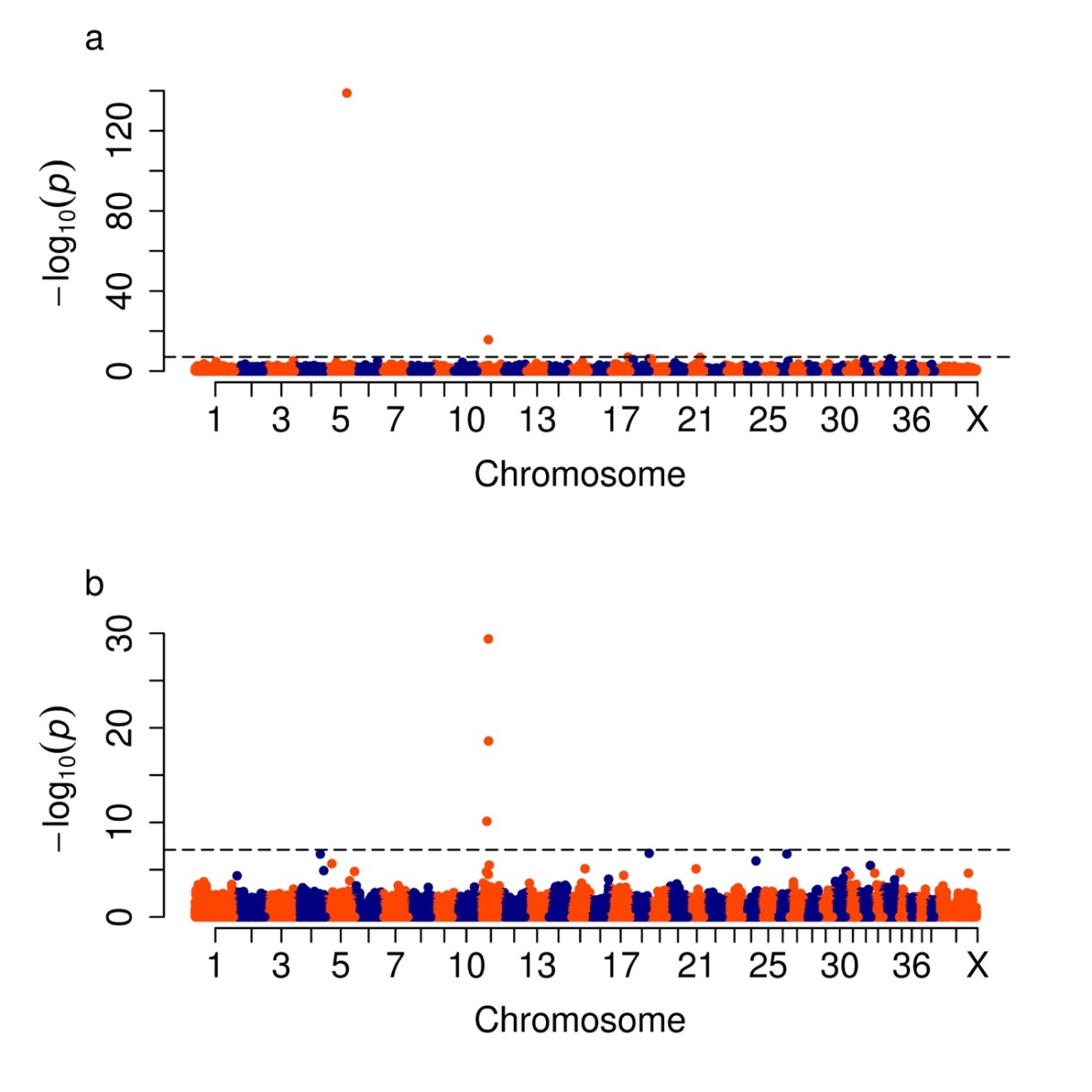

Supplement: S2 Fig — Dashed line is Bonferroni adjusted genome wide p < 0.01. (a) When GWAS was performed on a binary trait defined as yellow versus the others (black or chocolate), the SNP at CFA5:63,694,334 had the strongest association signal and is located within MC1R. (b) The tyrosine related protein 1 (TYRP1) gene, located at CFA11:33,317,110–33,336,030, causes chocolate coat color in black dogs so that when GWAS was performed on a binary trait defined as black versus chocolate, the marker at CFA11:33,326,685 was strongly associated. Note the Y axis scale denoting the strength of the association is different for the two color traits. (DOCX) [file pone.0176932.s002.docx]

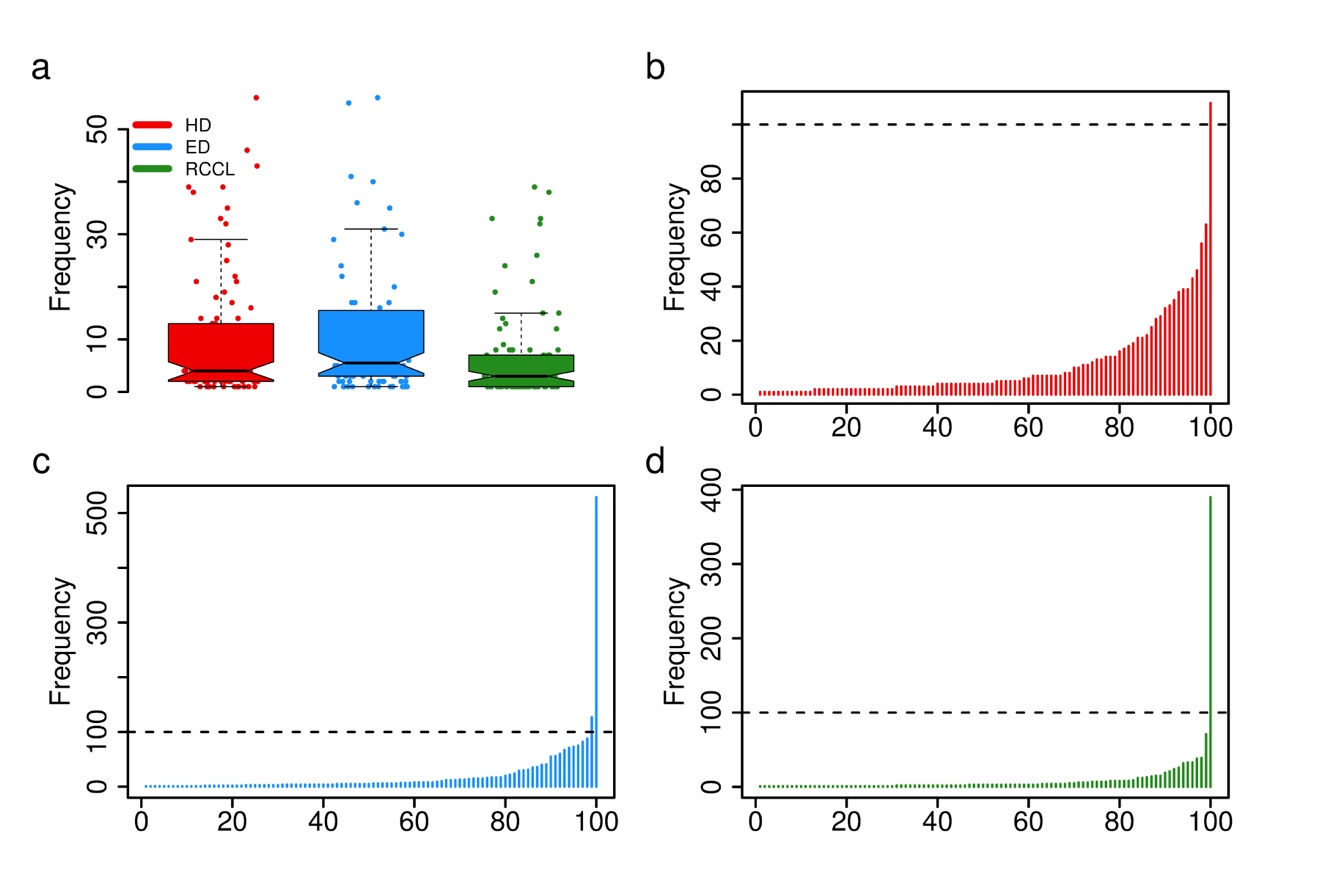

Supplement: S3 Fig — For the permutation, the real phenotype data of these three traits was shuffled and, for the resampling test, 80% of the individuals were sampled each time. This process was repeated 1,000 times and the most frequently associated loci were collected and their frequency displayed in the Box Plots (a) and the cumulative distributions for each trait (b, c, d). Black dashed line (b, c, d) refers to the cutoff of 100 that we used in real trait-genotype resample testing. Note the Y axis scale denoting the frequency of each replicated association is different for each panel. (DOCX) [file pone.0176932.s003.docx]

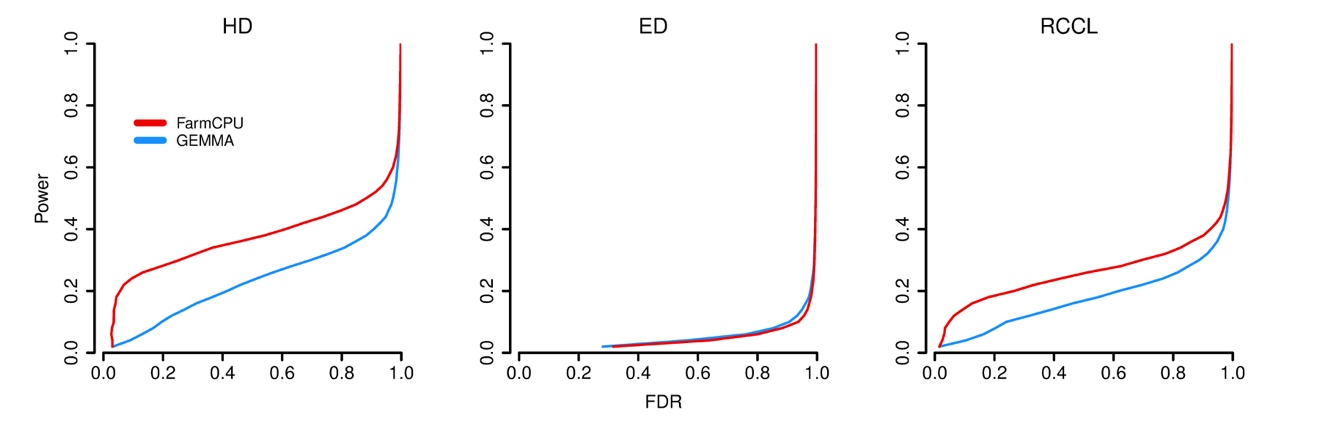
Figure S4

Supplement: S4 Fig — The vertical axis is the statistical power of 50 different randomly sampled causal loci and the horizontal axis is the false discovery rate (FDR) of different causal loci. The window size of each locus in both the power and FDR calculations was defined as 100,000 bp. (DOCX) [file pone.0176932.s004.docx]
