# Supplementary material for: A novel iterative mixed model to remap three complex orthopedic traits in dogs
Source: PLoS One. 2017 Jun 14;12(6):e0176932. doi: 10.1371/journal.pone.0176932 (PMC5470659; doi:10.1371/journal.pone.0176932)
Supplement: S1 Table — Hip dysplasia was measured as the Norberg angle. Elbow dysplasia was scored as present or absent based on physical examination and radiography, computed tomography and/or surgery. Rupture of the cranial cruciate ligament was assessed by physical examination, radiography, and surgery, and scored as control (unaffected, score of 1) or case, which could be a partial rupture (score of 2) or a complete rupture (score of 3) based on surgery. (DOCX) [file pone.0176932.s005.docx]

**Table S1**. Phenotype summary across breeds with the most individuals (or the remainder of the dogs) for hip dysplasia, elbow dysplasia, and rupture of the cranial cruciate ligament. Hip dysplasia was measured as the Norberg angle. Elbow dysplasia was scored as present or absent based on physical examination and radiography, computed tomography and/or surgery. Rupture of the cranial cruciate ligament was assessed by physical examination, radiography, and surgery, and scored as control (unaffected, score of 1) or case, which could be a partial rupture (score of 2) or a complete rupture (score of 3) based on surgery.

| Disease | Breed | Number of Dogs | Mean | Standard Deviation |
| --- | --- | --- | --- | --- |
| HipDysplasia | Labrador Retriever | 242 | 103.6 | 8.8 |
|  | Golden Retriever | 112 | 97.4 | 11.2 |
|  | German Shepherd Dog | 82 | 96.7 | 10.5 |
|  | English Setter | 79 | 104.6 | 4.8 |
|  | Newfoundland | 59 | 99.2 | 11.4 |
|  | Other | 347 | 103. 0 | 9.5 |
|  | All | 921 | 101.8 | 9.8 |
| Disease | Breed | N | Freq (controls) | Freq (cases) |
| Elbow Dysplasia | Labrador Retriever | 187 | 160 | 27 |
|  | Golden Retriever | 55 | 47 | 8 |
|  | German Shepherd Dog | 54 | 44 | 10 |
|  | English Setter | 79 | 27 | 52 |
|  | Newfoundland | 30 | 22 | 8 |
|  | Other | 341 | 308 | 33 |
|  | All | 746 | 633 | 113 |
| Disease | Breed | N | Mean | Standard Deviation |
| Rupture of the Cranial Cruciate Ligament | Labrador Retriever | 225 | 1.6 | 0.8 |
|  | Golden Retriever | 56 | 1.6 | 0.7 |
|  | German Shepherd Dog | 44 | 1.7 | 0.8 |
|  | Other | 278 | 1.4 | 0.7 |
|  | All | 603 | 1.5 | 0.7 |
